# Fatal Presumed Rhino-Orbito-Sinus Mucormycosis in a Child With Fanconi Anemia: A Case Report

**DOI:** 10.7759/cureus.108313

**Published:** 2026-05-05

**Authors:** Soufia Charaf, Sanaa Bouramdane, Kenza Elmkeddam, Sarra Benmiloud

**Affiliations:** 1 Pediatric Hematology and Oncology Department, Hassan II University Hospital, Fez, MAR; 2 Faculty of Medicine, Pharmacy and Dentistry, Sidi Mohamed Ben Abdellah University, Fez, MAR

**Keywords:** aplasia fanconi anemia, bone marrow, invasive fungal infection, mucormycosis, pediatric mucormycosis, rhino-orbito-sinus mucormycosis

## Abstract

We report the case of a 10-year-old male patient with bone marrow aplasia secondary to Fanconi anemia who was initially admitted for febrile purulent tonsillitis (no throat culture was performed before antibiotic initiation). Despite several courses of broad-spectrum antibiotic therapy, the clinical course was marked by persistent fever and progressive right-sided orbitofacial cellulitis. Imaging revealed extensive involvement of the right maxillary sinus with orbital extension. The appearance of a necrotic black palatal lesion (eschar) in the setting of profound immunosuppression is highly characteristic, if not pathognomonic, of invasive mucormycosis. Liposomal amphotericin B (5 mg/kg/day) was initiated; however, surgical debridement could not be performed due to severe thrombocytopenia (platelet count of 1,000/mm³). The patient subsequently developed septic deterioration requiring intensive care management and ultimately died. This report highlights the diagnostic challenges and the rapidly progressive course in a severely immunocompromised pediatric patient and emphasizes the importance of early recognition and prompt initiation of antifungal therapy, although management may remain limited in patients with profound cytopenia in whom surgical treatment is not feasible.

## Introduction

Mucormycosis is a rare but highly aggressive invasive fungal infection caused by filamentous fungi of the order Mucorales. It predominantly affects immunocompromised patients, particularly those with hematological malignancies, uncontrolled diabetes mellitus, severe neutropenia, or prolonged corticosteroid exposure. However, it remains exceptionally rare in children with Fanconi anemia [1]. Despite advances in antifungal therapy, mucormycosis remains associated with high morbidity and mortality (40-80%, depending on the site and underlying condition) due to its angioinvasive nature, leading to vascular thrombosis, tissue ischemia, and necrosis, which account for its rapid progression [1,2]. 

Mucormycosis can be divided into six types based on anatomic location: rhinocerebral, pulmonary, cutaneous, gastrointestinal, disseminated, and uncommon presentations (endocarditis, osteomyelitis, peritonitis, and pyelonephritis) [3]. Rhino-orbito-cerebral involvement represents one of the most common clinical forms, including rhino-orbito-sinus mucormycosis, which may rapidly extend from the paranasal sinuses to the orbit and surrounding facial structures [1]. Diagnosis remains challenging, particularly in pediatric and immunocompromised patients, due to nonspecific early symptoms and the rapid progression of the disease [1]. Early recognition and prompt initiation of antifungal therapy combined with surgical debridement, when feasible, are essential to improve survival, in accordance with current guidelines [1].

We report a case of invasive rhino-orbito-sinus mucormycosis in a child with bone marrow aplasia secondary to Fanconi anemia. This report highlights the diagnostic challenges and therapeutic limitations encountered in profoundly cytopenic patients in whom surgical management is not feasible.

## Case presentation

A 10-year-old male patient with bone marrow aplasia secondary to Fanconi anemia, receiving supportive care, including regular blood transfusions and iron chelation therapy, without curative treatment such as hematopoietic stem cell transplantation due to the absence of a suitable donor, was admitted for persistent fever associated with purulent exudative tonsillitis. On admission, the patient had a temperature of 39 °C, erythematous pultaceous tonsillitis, and odynophagia. Empirical intravenous antibiotic therapy with ceftriaxone and gentamicin was initiated due to suspected bacterial tonsillitis in the setting of febrile severe neutropenia, in accordance with principles for managing neutropenic sepsis.

Initial laboratory investigations revealed profound pancytopenia, with an absolute neutrophil count of 70/mm³, consistent with severe neutropenia (<500/mm³) and a high risk for invasive fungal infections, a platelet count of 1,000/mm³, and a hemoglobin level of 4.8 g/dL. A marked inflammatory response was observed, with C-reactive protein levels peaking at 416 mg/L. The remaining laboratory findings are summarized in Table 1.

**Table 1:** Laboratory findings CRP: C-reactive protein; PT: prothrombin time; aPTT: activated partial thromboplastin time

Parameters	Patient value	Unit	Reference range
Hemoglobin	4.8	g/dL	11–15
White blood cells	470	cells/mm³	4,000–10,000
Neutrophils	70	cells/mm³	1,500–8,000
Lymphocytes	280	cells/mm³	1,000–4,800
Monocytes	50	cells/mm³	200–800
Platelets	1,000	cells/mm³	150,000–450,000
CRP	416	mg/L	<5
Procalcitonin	32.36	ng/mL	<0.5
Creatinine	4	mg/L	3–10
Urea	0.17	g/L	0.15–0.45
Sodium	129	mmol/L	135–145
Potassium	2.9	mmol/L	3.5–5
Calcium	92	mg/L	85–105
Albumin	40.8	g/L	35–50
PT	56	%	70–100
aPTT	33	sec	25–35

**Figure 1:**
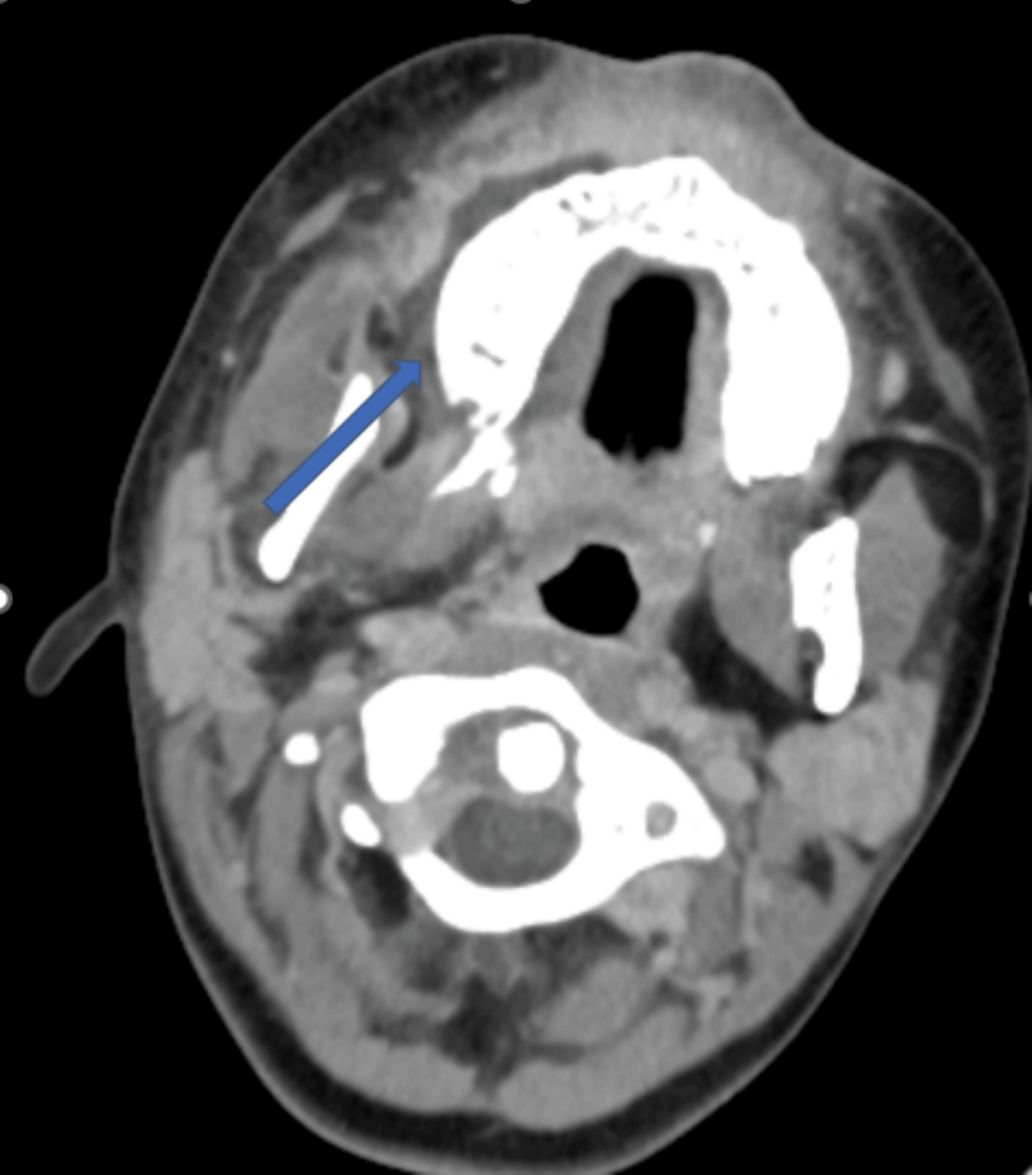
Axial CT image showing infiltration of the right jugal subcutaneous soft tissues (arrow) CT: computed tomography

After 24 hours, due to the persistence of fever, antibiotic therapy was escalated to ceftazidime, metronidazole, and amikacin. Because of a persistent febrile plateau, vancomycin was added after two days of hospitalization. Nasal endoscopic examination could not be performed due to the patient’s critical condition and severe thrombocytopenia. Two days later, whitish deposits appeared on the palate, raising suspicion of oropharyngeal candidiasis, and fluconazole therapy was initiated empirically with a loading dose of 12 mg/kg, followed by a maintenance dose of 6 mg/kg/day, before mucormycosis was considered. A right jugal swelling progressively developed and extended toward the palpebral and orbital regions, consistent with cervicofacial cellulitis. A cervicofacial CT scan was therefore performed and revealed marked phlegmonous infiltration of the right jugal subcutaneous soft tissues, extending to the lower eyelid and the intra- and extraconal orbital fat, without a well-defined collection (Figure 1).

The infiltration was more pronounced adjacent to the second lower premolar, beneath a deciduous molar with an enamel defect suggestive of dental caries, raising the possibility of an odontogenic portal of entry. Given the persistent fever and the progressive cervicofacial infection, antibiotic therapy was modified to piperacillin-tazobactam and teicoplanin. Blood cultures remained negative throughout hospitalization.

**Figure 2:**
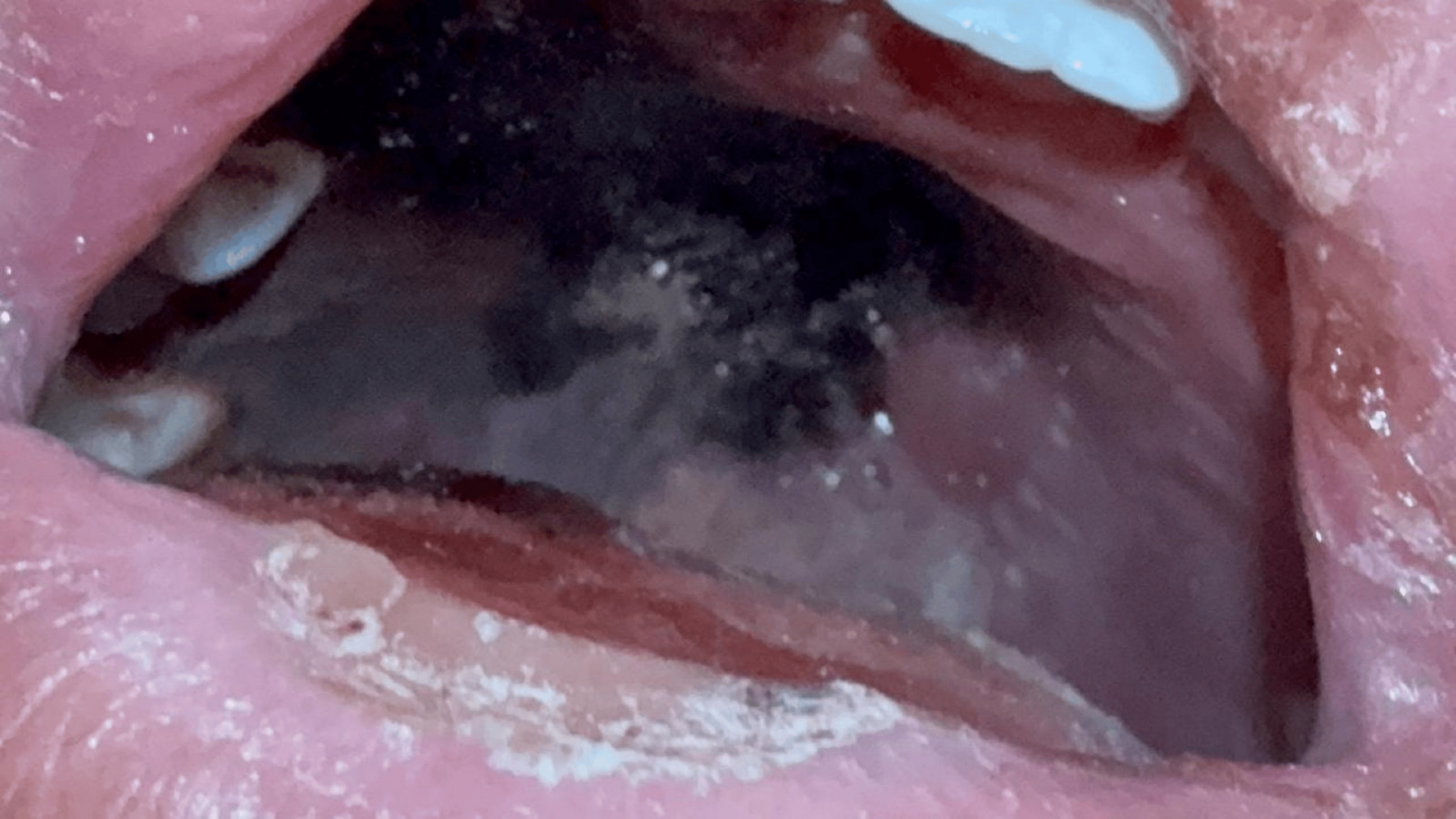
Black necrotic plaque of the hard palate suggestive of rhino-orbito-sinus mucormycosis

During the clinical course, the patient developed a necrotic blackish plaque on the hard palate, raising a strong suspicion of an invasive fungal infection, particularly mucormycosis (Figure 2). Polymerase chain reaction (PCR) testing for Mucorales on blood or a nasal swab was considered but could not be performed due to the local unavailability of the assay.

**Figure 3:**
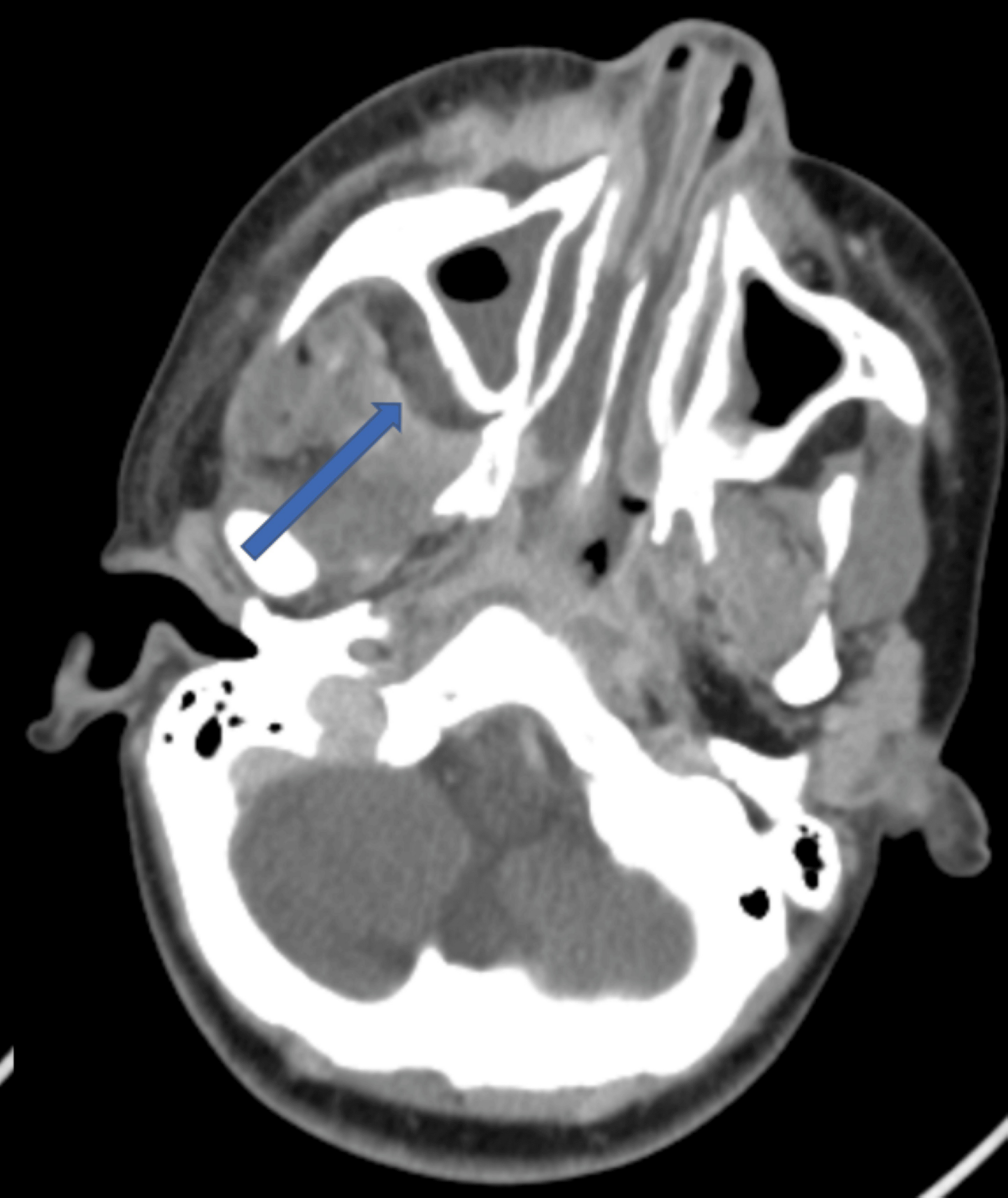
Axial CT image demonstrating opacification of the right maxillary and ethmoidal sinuses with extension of inflammatory infiltration toward the ipsilateral facial and orbital soft tissues (arrow) CT: computed tomography

**Figure 4:**
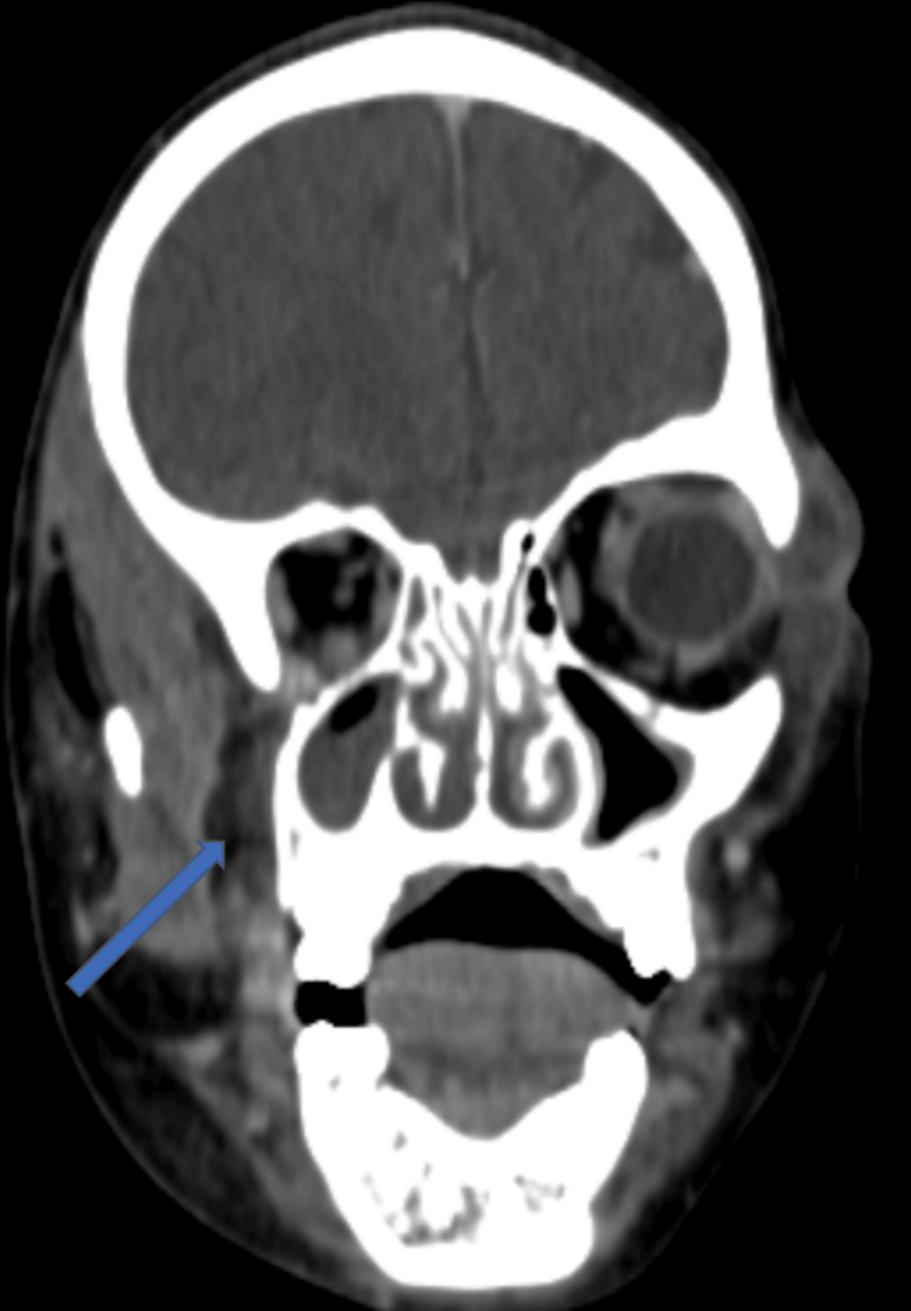
Coronal CT image showing right maxillary sinus opacification associated with infiltration of the intra- and extra-conal orbital fat (arrow) CT: computed tomography

Due to this suspicion, a follow-up cervicofacial and thoracic CT scan was performed. Imaging demonstrated circumferential thickening of the right maxillary sinus with hyperdense content and a collection within the right peri-antral fat measuring approximately 30 × 25 × 20 mm. This collection extended toward the infratemporal fossa via the sphenopalatine fissure and into the orbital region through the inferior orbital fissure with infiltration of the intra- and extraconal fat, resulting in grade I exophthalmos. Marked infiltration of the right facial subcutaneous tissues and parapharyngeal fat was also observed, associated with swelling of the ipsilateral masticatory muscles (Figures 3, 4).

Thoracic CT showed no pulmonary consolidation, nodules, pleural effusion, or the reversed halo sign (a finding associated with pulmonary mucormycosis but not pathognomonic) [1]. Given the clinical context of profound immunosuppression, the presence of a necrotic palatal eschar, and radiological findings consistent with invasive fungal sinusitis with orbital extension, rhino-orbito-sinus mucormycosis was strongly suspected. However, the diagnosis remained presumptive in the absence of microbiological or histopathological confirmation, as tissue biopsy could not be performed due to severe thrombocytopenia. Liposomal amphotericin B was initiated at a dose of 5 mg/kg/day. Surgical debridement could not be performed despite platelet transfusions, as the platelet count remained critically low (5,000/mm³), precluding safe surgical intervention.

During hospitalization, the patient developed respiratory distress and sepsis, requiring transfer to the ICU. Despite intensive supportive management, the clinical course was marked by progressive deterioration, leading to death after 23 days of evolution.

## Discussion

Epidemiology

Mucormycosis is a rare but severe invasive fungal infection with a heterogeneous epidemiological profile depending on geographic regions and underlying conditions [4]. Large registry data on 382 cases reported a male predominance of 61.8% with a median age of 48 years, while pediatric cases represented a minority [2]. In one cohort study, pediatric cases represented 7-16% of mucormycosis infections, with a mean age of approximately eight years and a male predominance [5]. Among clinical forms, rhino-orbito-cerebral mucormycosis (ROCM) is the most frequent presentation, accounting for 36.6% of reported cases in international registry analyses. This entity represents a clinical spectrum ranging from localized sinonasal infection to advanced disease with orbital and intracranial extension. Within this group, localized rhino-sinus involvement accounts for about 35% of cases, while rhino-orbital involvement represents 39.3% [2]. Pulmonary involvement is less common in the general pediatric population but becomes more prevalent in children with malignancies [5].

Regarding etiological agents, Rhizopus species are the most frequently identified pathogens in pediatric mucormycosis. Other genera, including Mucor spp. (0-53%), Cunninghamella spp. (0-28%), and Lichtheimia spp. (4-16%) have also been reported, with geographic variability in species distribution [5]. The distribution of underlying conditions differs across regions: diabetes mellitus remains the most common predisposing factor worldwide, particularly in low- and middle-income countries, representing approximately 25% of cases, whereas hematological malignancies are the leading risk factor in children, accounting for 16-52% of pediatric cases [5]. In addition to systemic risk factors, local factors such as dental infections have been reported in association with mucormycosis in pediatric patients, highlighting the importance of considering this diagnosis in immunocompromised children presenting with odontogenic lesions [5].

Aplastic anemia is rarely reported, representing approximately 1.7% of cases in registry data [2]. Despite its low prevalence, it is associated with a high risk of severe infections and poor outcomes, particularly in the setting of invasive fungal infections such as mucormycosis, where mortality remains extremely high in profoundly immunosuppressed patients [6]. In this context, aplastic anemia was the main predisposing factor, and the clinical presentation corresponded to rhino-orbito-sinus mucormycosis. This was the only pediatric case of mucormycosis diagnosed in our department during the study period from 2019 to 2026.

Clinical presentation 

Rhino-orbito-sinus mucormycosis usually originates from the paranasal sinuses and may extend to the orbit and brain, leading to facial edema, ocular pain, and necrotic lesions such as palatal ulcers or black eschars [1]. In one cohort, the most common symptoms were orbital pain or swelling (27.4%), nasal discharge (18.3%), headache (17.1%), facial pain (15.9%), and fever (5.5%), while palatal ulceration was less frequent (2.4%) [7,8]. Some clinical manifestations may represent warning signs of progression toward invasive rhino-orbito-cerebral disease, including cranial nerve palsy, diplopia, and proptosis [9]. Pulmonary mucormycosis generally presents with fever, cough, dyspnea, chest pain, and sometimes hemoptysis, with imaging findings such as nodules, consolidation, or cavitary lesions. Cutaneous forms typically occur after trauma or skin disruption and present with necrotic lesions or eschar formation. Gastrointestinal mucormycosis remains rare and usually manifests with nonspecific symptoms such as abdominal pain or distension [1,8]. Similarly, our patient presented with clinical features consistent with those described in the literature, including facial edema, orbital involvement, and necrotic palatal lesions, findings suggestive of rhino-orbito-sinus mucormycosis.

Diagnosis 

The diagnosis of mucormycosis remains challenging and requires a combination of clinical suspicion, imaging findings, and microbiological or histopathological confirmation [1]. Early diagnosis is essential because of the rapid progression of the infection and its high mortality [1,6]. Imaging plays an important role in the initial evaluation. In rhino-orbito-cerebral forms, cranial CT or MRI is recommended to detect sinus involvement and evaluate extension to orbital or intracranial structures, with MRI providing greater sensitivity for soft-tissue and central nervous system involvement [1]. In suspected pulmonary mucormycosis, chest CT may reveal consolidation, nodules, pleural effusion, or the reversed halo sign [1,8]. In addition, imaging is essential to assess the extent of disease, and cranial, thoracic, and abdominal imaging may be required, particularly in patients with underlying malignancies. Given the rapid progression of mucormycosis, repeat imaging may be necessary, especially in unstable patients [1].

Definitive diagnosis relies on histopathological examination and microbiological confirmation [1]. Histopathology contributes to diagnosis in approximately 62.6% of cases, while direct microscopy (e.g., KOH mount of a palatal swab) is positive in up to 97.3% and cultures in 82% [1,8]. In our case, neither was performed due to the absence of an accessible specimen and the patient's bleeding risk. Typical histological features include broad, ribbon-like, irregular hyphae that are non-septate or sparsely septate, often associated with angioinvasion and tissue necrosis [2,8]. Although culture allows identification at the genus or species level, negative cultures may occur despite compatible histological findings, and blood cultures are usually non-contributive due to the angioinvasive nature of Mucorales [10]. Molecular diagnostic methods such as PCR and metagenomic next-generation sequencing have recently emerged as useful tools for pathogen identification, particularly when conventional microbiological techniques are inconclusive [8,10].

In the present case, the diagnosis was established based on clinical suspicion, the underlying condition, and radiological findings. Craniofacial CT scan findings were suggestive of rhino-orbito-sinus mucormycosis, while a chest CT scan was performed as part of the extension workup to search for pulmonary mucormycosis, which was not detected. Histopathological confirmation could not be performed because of severe thrombocytopenia, and PCR testing was not available locally, representing a major diagnostic limitation of this case.

Treatment

The effective management of mucormycosis relies on a rapid multimodal approach combining early antifungal therapy, surgical debridement whenever feasible, and correction of underlying predisposing factors. Because of the aggressive and rapidly progressive nature of the infection, treatment should be initiated as soon as mucormycosis is suspected, without delaying therapy while awaiting diagnostic confirmation, as recommended by current guidelines [1]. Surgical resection or debridement is recommended whenever possible in combination with systemic antifungal therapy. However, the feasibility of surgery depends on the patient’s clinical condition and the anatomical extent of the disease [1,8]. Treatment of mucormycosis in neonates, children, and adolescents is largely based on adult observational data and pediatric pharmacokinetic and safety studies, with lipid formulations of amphotericin B recommended as first-line therapy [11].

Liposomal amphotericin B is most commonly used, typically administered at doses of 5-10 mg/kg/day, with higher doses considered in cases involving the central nervous system. Amphotericin B deoxycholate is effective, but its use is limited by toxicity and is generally reserved for settings where lipid formulations are unavailable. Isavuconazole and posaconazole represent alternative options and may be used as first-line or salvage therapy, particularly in patients who cannot tolerate amphotericin B [1,2]. There are cases of successful treatment of mucormycosis caused by Rhizopus arrhizus with amphotericin B and itraconazole [12]. Registry data indicate that nearly half of patients receive combined surgical and antifungal treatment, whereas others are managed with antifungal therapy alone based on disease severity and clinical constraints [2]. 

Treatment strategies in pediatric mucormycosis are largely extrapolated from adult studies, as pediatric-specific data remain limited and are mainly derived from case reports and small case series, highlighting an important gap in current research [7]. Nevertheless, therapeutic principles remain consistent across pediatric age groups and rely on the urgent initiation of antifungal therapy, surgical debridement whenever feasible, and management of underlying risk factors [2,8,13]. In our case, liposomal amphotericin B was initiated; however, surgical management was not feasible due to severe thrombocytopenia. Less invasive interventions, such as transnasal endoscopic inspection or local antifungal irrigation, were considered but deemed unlikely to alter outcomes given the extensive disease burden.

Outcome and prognosis

Mucormycosis remains a highly lethal invasive fungal infection, with overall mortality rates ranging from 40% to 80% depending on the site of infection and underlying condition. In pediatric populations, mortality varies from 33.3% to 56%, with global estimates of approximately 46%. Disseminated disease carries the worst prognosis, with mortality reaching approximately 79.4%, while central nervous system involvement exceeds 80%, whereas localized forms such as sinus disease may have better outcomes when early diagnosis and combined medical-surgical management are achieved [1,2]. In our patient, the fatal outcome was multifactorial, related to the presence of severe bone marrow failure with profound immunosuppression and neutropenia, the inability to perform surgical debridement due to severe thrombocytopenia, and the rapidly progressive nature of the infection, conditions recognized as major risk factors for invasive fungal infections and poor prognosis.

The patient is deceased, and all identifying information has been removed to ensure anonymity.

## Conclusions

This report highlights the importance of maintaining a high index of suspicion for rhino-orbito-sinus mucormycosis in neutropenic children presenting with persistent fever and rapidly progressive cervicofacial infection. Early recognition and prompt initiation of antifungal therapy are crucial, and management requires a multidisciplinary approach. In our patient, management was limited by severe cytopenia, which prevented surgical intervention.
